# *Col4a2* Mutations Contribute to Infantile Epileptic Spasm Syndrome and Neuroinflammation

**DOI:** 10.7150/ijms.97164

**Published:** 2024-07-02

**Authors:** Chunhui Hu, Deying Liu, Hua Wang

**Affiliations:** 1 Department of Neurology, Fujian Children's Hospital, College of Clinical Medicine for Obstetrics & Gynecology and Pediatrics, Fujian Medical University, Fuzhou, China.; 2 College of Clinical Medicine for Obstetrics & Gynecology and Pediatrics, Fujian Medical University, Fuzhou, China.; 3 Department of Pediatrics, Shengjing Hospital of China Medical University, Shenyang, China.

**Keywords:** *Col4a2*, infantile epileptic spasm syndrome, neuroinflammation, astrocytes

## Abstract

There are more than 70 million people worldwide living with epilepsy, with most experiencing the onset of epilepsy in childhood. Despite the availability of more than 20 anti-seizure medications, approximately 30% of epilepsy patients continue to experience unsatisfactory treatment outcomes. This situation places a heavy burden on patients' families and society. Childhood epilepsy is a significant chronic neurological disease that is closely related to genetics. *Col4a2*, the gene encoding the α2 chain of type IV collagen, is known to be associated with multiple diseases due to missense mutations. The *Col4a2* variant of collagen type IV is associated with various phenotypes, including prenatal and neonatal intracranial hemorrhage, porencephaly, porencephaly with cataracts, focal cortical dysplasia, schizencephaly, strokes in childhood and adolescence, and sporadic delayed hemorrhagic stroke. Although epilepsy is recognized as a clinical manifestation of porencephaly, the specific mechanism of *Col4a2*-related epileptic phenotypes remains unclear. A total of 8 patients aged 2 years and 2 months to 18 years who were diagnosed with *Col4a2*-related infantile epileptic spasm syndrome were analyzed. The seizure onset age ranged from 3 to 10 months. Initial EEG results revealed hypsarrhythmia or multiple and multifocal sharp waves, spike waves, sharp slow waves, or spike slow waves. Elevated levels of the cytokines IL-1β (32.23±12.58 pg/ml) and IL-6 (45.12±16.03 pg/ml) were detected in the cerebrospinal fluid of these patients without any signs of infection. Following antiseizure treatment, decreased IL-1β and IL-6 levels in the cerebrospinal fluid were noted when seizures were under control. Furthermore, we aimed to investigate the role of *Col4a2* mutations in the development of epilepsy. Through the use of immunofluorescence assays, ELISA, and Western blotting, we examined astrocyte activity and the expression of inflammatory cytokines such as IL-1β, IL-6, and TNF-α after overexpressing an unreported *Col4a2* (c.1838G>T) mutant in CTX-TNA cells and primary astrocytes. We found that the levels of the inflammatory factors IL-1β, IL-6, and TNF-α were increased in both CTX-TNA cells (ELISA: p = 0.0087, p<0.001, p<0.001, respectively) and primary astrocytes (ELISA: p = 0.0275, p<0.001, p<0.001, respectively). Additionally, we conducted a preliminary investigation of the role of the JAK/STAT pathway in *Col4a2* mutation-associated epilepsy. Col4a2 mutation stimulated astrocyte activation, increasing iNOS, COX-2, IL-1β, IL-6, and TNF-α levels in both CTX-TNA cells and primary astrocytes. This mutation also activated the JAK/STAT signaling pathway, leading to increased phosphorylation of JAK2 and STAT3. Treatment with the JAK/STAT inhibitor WP1066 effectively counteracted this effect in primary astrocytes and CTX-TNA cells. To date, the genes who mutations are known to cause developmental and epileptic encephalopathies (DEEs) are predominantly grouped into six subtypes according to function. Our study revealed that an unreported mutation site Col4a2^Mut^ (c.1838G>T) *of which can cause* neuroinflammation, may be a type VII DEE-causing gene.

## Introduction

Epilepsy is a common neurological disease that affects approximately 70 million individuals globally 1. The causes of epilepsy are categorized as infectious, immune, structural, metabolic, genetic, or unknown factors. Genetic factors are increasingly recognized as significant contributors to childhood epilepsy. The process of epileptogenesis and epilepsy progression includes neuronal damage, heightened oxidative stress, abnormal glial cell activity, mitochondrial dysfunction, and inflammatory responses 2.

The immune-inflammatory response has gained increasing recognition as a significant factor in the development and progression of epilepsy. Various inflammatory factors can contribute to the activation of signaling pathways in neurons, microglia, and astrocytes, leading to alterations in synaptic transmission and plasticity and ultimately contributing to the pathogenesis of epilepsy 3. A recent study identified neuroinflammation as the pathological signature in genetic epilepsy 4. Additionally, interleukin-1 receptor accessory protein-like 1 (IL1RAPL1), a member of the IL-1 receptor family, is selectively expressed in excitatory synapses of the brain. Mutations in the gene encoding IL1RAPL1 have been linked to intellectual disabilities, autism spectrum disorders, and epilepsy 5. The proinflammatory cytokine IL-1β, which is produced and released by activated microglia and astrocytes, targets specific pathways that can impair neuronal plasticity and affect synaptic structure and function. Notably, IL1RAPL1, type 2 K+-Cl cotransporter protein (KCC2), and methylCpG binding protein 2 (MECP2) are the critical proteins affected by this process, establishing a connection between inflammation and genetic synapses 5-7. Pathogenic mutation of *MECP2* is associated with Rett syndrome. Studies have indicated that immune system damage linked to *MECP2* mutations can drive disease progression by altering microglia, triggering persistent subclinical inflammation in the peripheral blood, and causing excessive oxidative stress 8, 9. In females with *PCDH19* gene mutation-related epilepsy, corticosteroids have shown therapeutic effects when used as an adjuvant treatment, suggesting that inflammation plays a role in the development of *PCDH19* epilepsy 10. Patients with *CDKL5* deficiency also exhibit subclinical chronic inflammation, as indicated by plasma parameters 11. In individuals with tuberous sclerosis, a notable increase in complement C3 expression was observed in the white and gray matter of the lesion after surgical resection. Complement factors are colocalized with astrocytes, microglia, and neurons, indicating that significant activation of the complement pathway is crucial for tuberous sclerosis pathogenesis 12. These findings highlight the significance of neuroinflammation in the development of hereditary epilepsy, suggesting that targeting inflammation could be a promising treatment approach.

Immune dysfunction may be involved in neuroinflammation, leading to the development of epilepsy. Increased inflammatory mediator expression can trigger inflammatory signaling pathways in neurons and glial cells, potentially affecting the blood‒brain barrier and contributing to epileptogenesis.* Col4a1* and *Col4a2*, which encode the α1 and α2 chains of type IV collagen, respectively, are crucial basement membrane components 13-15. Previous studies have linked missense mutations in Col4a1 and Col4a2 to various diseases, including ischemic cerebral lesions and cortical development malformations 16-19. For example, Col4a1 and Col4a2 gene mutations were detected in patients with ischemic cerebral lesions. Mutations in Col4a2 have been associated with cortical development malformations, while Col4a1 mutations have been linked to perinatal cerebral hemorrhage and porencephaly 20. Epilepsy resulting from mutations in the *Col4a2* gene is often caused by de novo mutations. In our study, we identified a child with epilepsy who carried the same *Col4a2* mutation as his mother. Interestingly, the child's mother did not exhibit epilepsy but only experienced mental retardation. This observation led us to consider the possibility of incomplete penetrance associated with the *Col4a2* gene mutation. Subsequently, we focused our validation efforts on the c.1838G>T site. Col4a2 encodes type IV collagen, which forms the basement membrane and affects the permeability of the blood‒brain barrier. Astrocytes, a crucial component of the blood‒brain barrier, are involved in the secretion of inflammatory molecules. Therefore, we speculated that Col4a2 mutations may affect the function of astrocytes.

In this study, we retrospectively analyzed eight patients who were initially diagnosed with *Col4a2*-related infantile epileptic spasm syndrome and discovered elevated proinflammatory cytokine levels in the cerebrospinal fluid. Then, we established an LPS-induced astrocyte model to evaluate the effects of the *Col4a2* mutation on epilepsy progression. We detected aberrant activation and upregulation of proinflammatory factors in both CTX-TNA cells and primary astrocytes after *Col4a2* mutation. Our findings may provide novel therapeutic strategies for treating genetic epilepsy.

## Materials and Methods

### Patients

Eight patients who were diagnosed with *Col4a2*-related infantile epileptic spasm syndrome in the Department of Neurology, Fujian Children's Hospital, between June 2021 and March 2024 were included in this retrospective study. This human study was approved by the Ethics Committee of Fujian Children's Hospital (2023ETKLR11006). The animal experiments were approved by the Experimental Animal Ethics Committee of Fujian Medical University (IACUC FJMU 2024-0016). The study was performed following the Declaration of Helsinki. Patients with epilepsy caused by intrauterine infection, perinatal brain injury, metabolic disease, or significant brain structural abnormalities were not included in the study. This study focused on various parameters related to the epilepsy phenotype, such as seizure onset age, seizure semiology, intelligence, electroencephalography (EEG), brain magnetic resonance imaging (MRI) findings, cerebrospinal fluid cytokine levels, clinical genetic evaluations, and seizure outcomes. Trio-based whole-exome sequencing was conducted on peripheral blood samples from eight patients to identify pathogenic mutations. The pathogenicity of variants was assessed based on ACMG guidelines. The parental origin of the variants was confirmed using PCR Sanger sequencing, and causal mutations were identified accordingly. Seizure control was defined as a lack of seizures for three times longer than the maximum interseizure interval or one year. Seizure outcome was evaluated using the Engel grading system, with assessments conducted every six months through inpatient, outpatient, or telephone follow-ups.

### Animals

Sprague-Dawley (SD) rats weighing 200 to 220 g were obtained from Vital River Laboratory (China). All animals were raised under specific pathogen-free conditions on a normal 12-h light-dark cycle. The environment was maintained at a temperature of 22-25 °C and a humidity between 40% and 70%. The rats had ad libitum access to water and rodent food. All rats were deeply anesthetized with isoflurane and euthanized by cervical dislocation.

### Isolation of primary astrocytes

Brains were dissected from 1-day-old rats, and the cerebral cortex was removed. The tissue was then cut into pieces and digested with 0.05% trypsin at 37 °C for 10 minutes, and this process was repeated twice. The supernatant was aspirated, and the remaining cells were passed through a 70 μm sieve and centrifuged at 300 × g for 5 minutes. The resulting supernatant was discarded. The cells were then resuspended in complete medium, centrifuged at 200 × g for 5 minutes, and resuspended again. These cells were seeded in culture flasks and grown until they reached 90% confluence. When the culture reached near confluence, adherent cells were detached using trypsin, centrifuged at 200 × g for 5 minutes, and resuspended in complete medium before being seeded onto culture flasks or plates coated with polylysine for subculture or group experiments.

### Cell culture and treatment

The CTX-TNA rat astrocyte cell line was obtained from iCell (China) and cultured following the supplier's instructions. The cell lines tested negative for mycoplasma. The cells were cultured in DMEM (Gibco, USA) supplemented with 10% FBS (Gibco, USA) and 1% penicillin‒streptomycin (SolarBio, China) at 37 °C with 5% CO_2_. The experimental groups included the control group (no drug treatment), LPS group (cells stimulated with 5 μg/mL LPS for 48 hours to induce neuroinflammation), LPS+NC group (cells stimulated with LPS and transfected with an empty adenoviral vector), LPS+*Col4a2^Wt^* group (cells stimulated with LPS and transfected with the *Col4a2^Wt^*-AdV vector), LPS+Col4a2*^Mut^* group (cells stimulated with LPS and transfected with the *Col4a2^Mut^*-AdV vector carrying the c.1838G>T, p.G613V mutation), and LPS+*Col4a2^Mut^*+WP1066 group (cells treated with the JAK2/STAT inhibitor WP1066 (5 μM, HY-15312; MCE, China) after stimulation with LPS and transfection with the Col4a2*^Mut^*-AdV vector).

### Immunofluorescence (IF) staining

Cells fixed in 4% paraformaldehyde were treated with 0.1% Triton X-100 for 15 minutes, followed by blocking in 5% bovine serum albumin (BSA) for 1 hour. Subsequently, the cells were incubated with a primary antibody specific for GFAP for two hours at room temperature (RT) and then with a secondary antibody (Cy3-conjugated anti-rabbit IgG; Proteintech, SA00009-2) at 37 °C for 1 hour. Finally, staining with DAPI (C1006; Beyotime, China) was performed, and observation was carried out via confocal laser microscopy (Leica, TCS SP8). The fluorescence intensity in each image was quantified using ImageJ software. The fluorescence intensity of GFAP was measured in 5 different groups, with 50 CTX-TNA cells per group from 3 repeated experiments.

### ELISA

The cell culture medium was centrifuged at 2000 × g for 10 minutes to remove debris, and the cell culture supernatants were collected for analysis. The levels of IL-1β, IL-6, and TNF-α were assessed using a Rat IL-1 beta ELISA Kit (ab255730; Abcam, USA), a Rat IL-6 ELISA Kit (ab234570; Abcam, USA), and a Rat TNF alpha ELISA Kit (ab46070; Abcam, USA) following the manufacturer's instructions with 3 biological replicates per group. The optical density at 450 nm was measured using a microplate reader, and concentrations of each cytokine were calculated based on their respective standard curves.

### Western blotting

Cellular protein extracts were isolated using RIPA buffer (Beyotime, China), and electrophoresis was performed. Following transfer to PVDF membranes, the protein samples were incubated with primary antibodies overnight at 4 °C. The primary antibodies and secondary antibody were diluted with primary antibody diluent and secondary antibody diluent, respectively. These antibodies included anti-GFAP (ab7260; Abcam, USA), anti-iNOS (ab178945; Abcam, USA), anti-COX-2 (ab179800; Abcam, USA), anti-IL-1β (ab254360; Abcam, USA), anti-IL-6 (ab9324; Abcam, USA), anti-TNF-α (ab307164; Abcam, USA), anti-JAK2 (ab108596; Abcam, USA), anti-p-JAK2 (44-426G; Thermo, USA), anti-STAT3 (ab68153; Abcam, USA), anti-p-STAT3 (ab32143; Abcam, USA), and anti-Actin (3700; CST, USA) antibodies. Subsequently, the membrane was rinsed three times with TBST and incubated with diluted secondary antibody for one hour at room temperature. Following three additional washes in TBST, the membrane was visualized using an enhanced chemiluminescence (ECL) detection system.

### Statistical analysis

Statistical analyses were conducted using GraphPad Prism 8 (San Diego, California, USA). The data are shown as the means ± standard errors. Differences between groups were evaluated using one-way ANOVA. A *P* value < 0.05 was considered to indicate statistical significance.

## Results

### Patient demographics

To explore the role of *Col4a2* mutations in epileptogenesis, we first conducted a retrospective analysis of eight patients who were diagnosed with *Col4a2*-related infantile epileptic spasm syndrome. A total of 8 patients—six males and two females with ages ranging from 2 years and two months to 18 years—were analyzed (Table 1). The age of seizure onset ranged from 3 months to 10 months. None of the patients were closely related, and their parents were unrelated. The pregnancy history and delivery were uneventful for all patients. In the family history, only patient 7's mother had moderate mental retardation without epilepsy. 6 patients showed epileptic spasms. Patient 5 and patient 6 showed epileptic spasms, focal seizures, and tonic seiures. Initial EEG findings showed hypsarrhythmia or multiple and multifocal sharp waves, spike waves, sharp slow waves, or spike slow waves. Patient 3 and patient 8's EEGs were later normalized. Genetic analysis revealed eight missense mutations related to *Col4a2* infantile epileptic spasm syndrome, seven of which are likely pathogenic and the other one is of uncertain significance (Table 2). Cerebrospinal fluid cytokine levels were measured in 8 patients before and after anti-seizure treatment. Cerebrospinal fluid cytokine levels were elevated in 6 patients without infection before they became seizure free, and we detected increased IL-1β and IL-6 levels in these patients (Table 3). However, peripheral blood cytokine levels were normal. Following anti-seizure treatment, cytokine levels in the cerebrospinal fluid decreased in 4 patients whose seizures were controlled but remained elevated in patients with uncontrolled seizures (Table 4). Four patients whose cytokine levels decreased at the final follow-up remained seizure-free. Our clinical data showed that proinflammatory cytokine levels are elevated in the cerebrospinal fluid of patients with *Col4a2* mutation-associated epilepsy, whereas the levels of these proinflammatory cytokines can be reduced after effective treatment.

### *The Col4a2* (c.1838G>T, p.G613V) mutation promotes astrocyte activation *in vitro*

**Figure 1:**
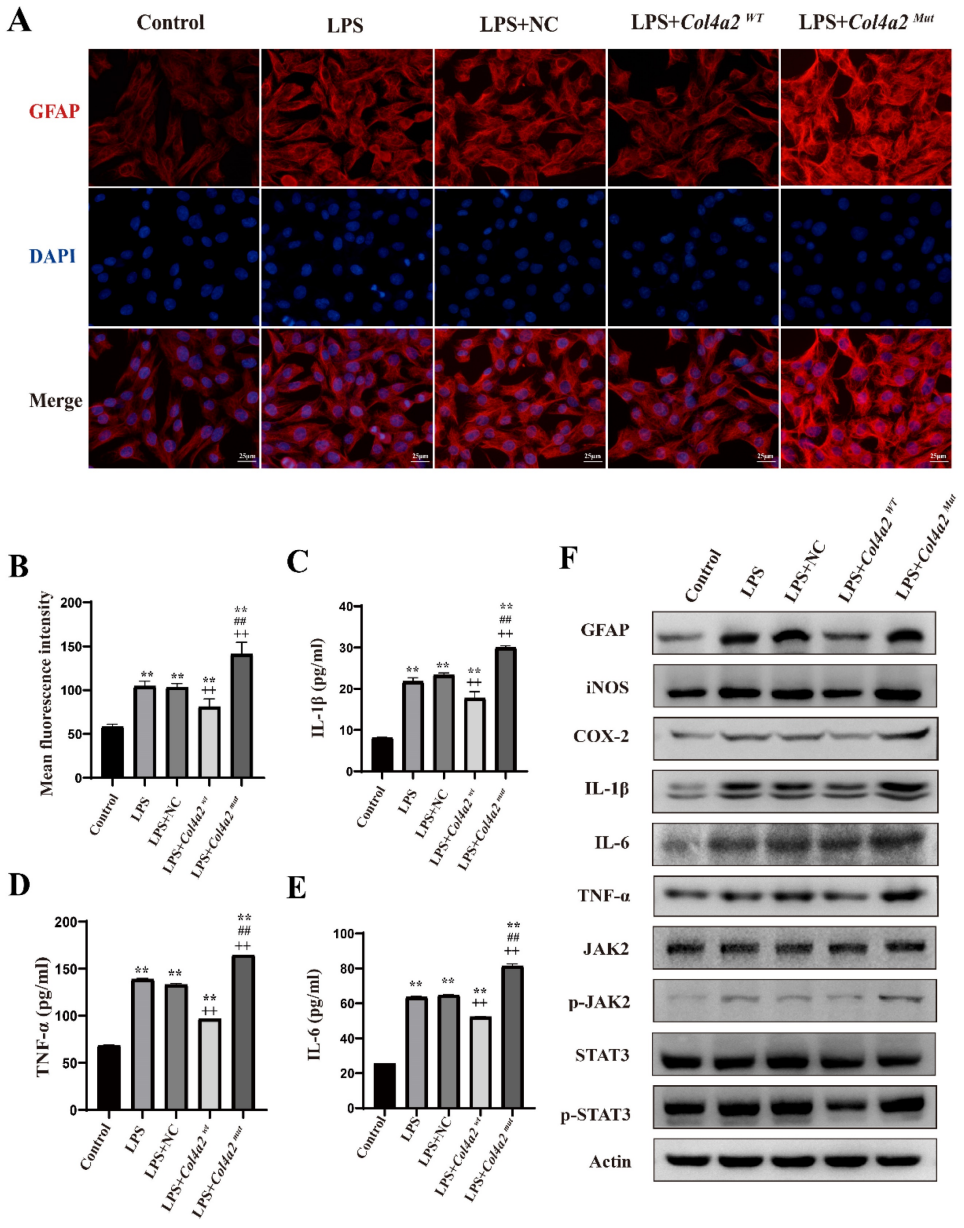
** Mutation of *Col4a2* promotes inflammation and astrocyte activation in CTX-TNA cells.** CTX-TNA cells were stimulated with LPS and transfected with the *Col4a2^Wt^*-AdV vector or the *Col4a2^Mut^*-AdV vector. (A) The fluorescence intensity of GFAP (red) was measured in 5 different groups, with 50 CTX-TNA cells per group from 3 repeated experiments. The scale bar represents 25 μm. (B) Quantitative graphs displaying the mean fluorescence intensity of the GFAP-positive cells in (A). (C-E) Enzyme-linked immunosorbent assays were used to quantify IL-1β, IL-6, and TNF-α levels in the supernatant of CTX-TNA cells from 3 repeated experiments. The figure includes results from one-way ANOVA with calculated *p* values. (F) Western blot analysis was performed for GFAP, IL-1β, IL-6, TNF-α, JAK2, p-JAK2, STAT3, and p-STAT3. The proteins for the western blot analysis were extracted from cell lysates. **: vs. con, p<0.01; ++: vs. LPS+NC, p<0.01; ##: vs. LPS+ *Col4a2^Wt^*, *p*<0.01.

To assess the impact of *Col4a2* gene mutations and LPS treatment on CTX-TNA cells and primary astrocytes, we examined GFAP expression using IF and WB. Our results revealed a significant increase in GFAP expression following *Col4a2* mutation in both CTX-TNA cells (IF: *p*<0.001; Fig. 1A, B, F) and primary astrocytes (IF: *p* = 0.0017; Fig. 3A, B, F), indicating increased astrocyte activity. Conversely, overexpression of *Col4a2^Wt^* reduced GFAP expression in both CTX-TNA cells (IF: *p*<0.001; Fig. 1A, B, F) and primary astrocytes (IF: *p*<0.001; Fig. 3A, B, F), suggesting a potential protective role of the *Col4a2^Wt^* gene against excessive astrocyte activation.

### *Col4a2* mutation promotes inflammatory responses in astrocytes

Following the discovery of excessive astrocyte activation, we further analyzed inflammatory cytokines, specifically iNOS, COX2, IL-1β, IL-6, and TNF-α. The expression of COX-2 and iNOS was found to be induced by the *Col4a2* mutation in both CTX-TNA cells (Fig. 1C-F) and primary astrocytes (Fig. 3C-F). Furthermore, the levels and secretion of IL-1β, IL-6, and TNF-α were consistently increased by the *Col4a2* mutation in both CTX-TNA cells (ELISA: *p* = 0.0087, *p*<0.001, *p*<0.001, respectively; Fig. 1C-F) and primary astrocytes (ELISA: *p* = 0.0275, *p*<0.001, *p*<0.001, respectively; Fig. 3C-F).

Interestingly, we observed that *Col4a2^Wt^* was able to weaken the inflammatory response induced by *Col4a2^Mut^*. The levels of the aforementioned inflammatory cytokines were significantly decreased when *Col4a2^Wt^* was transfected into both CTX-TNA cells (ELISA: IL-1β, *p*<0.001; IL-6, *p*<0.001; TNF-α, *p*<0.001; Fig. 1C-F) and primary astrocytes (ELISA: IL-1β, *p* = 0.0428; IL-6, *p*<0.001; TNF-α,* p*<0.001; Fig. 3C-F). These findings suggest that excessive astrocyte activation can trigger inflammatory responses. Mutation of *Col4a2* enhanced the secretion of inflammatory cytokines, while overexpression of *Col4a2^Wt^* protected against inflammation.

### *Col4a2* mutation promotes astrocyte activation and inflammatory responses by stimulating JAK/STAT signaling

**Figure 2:**
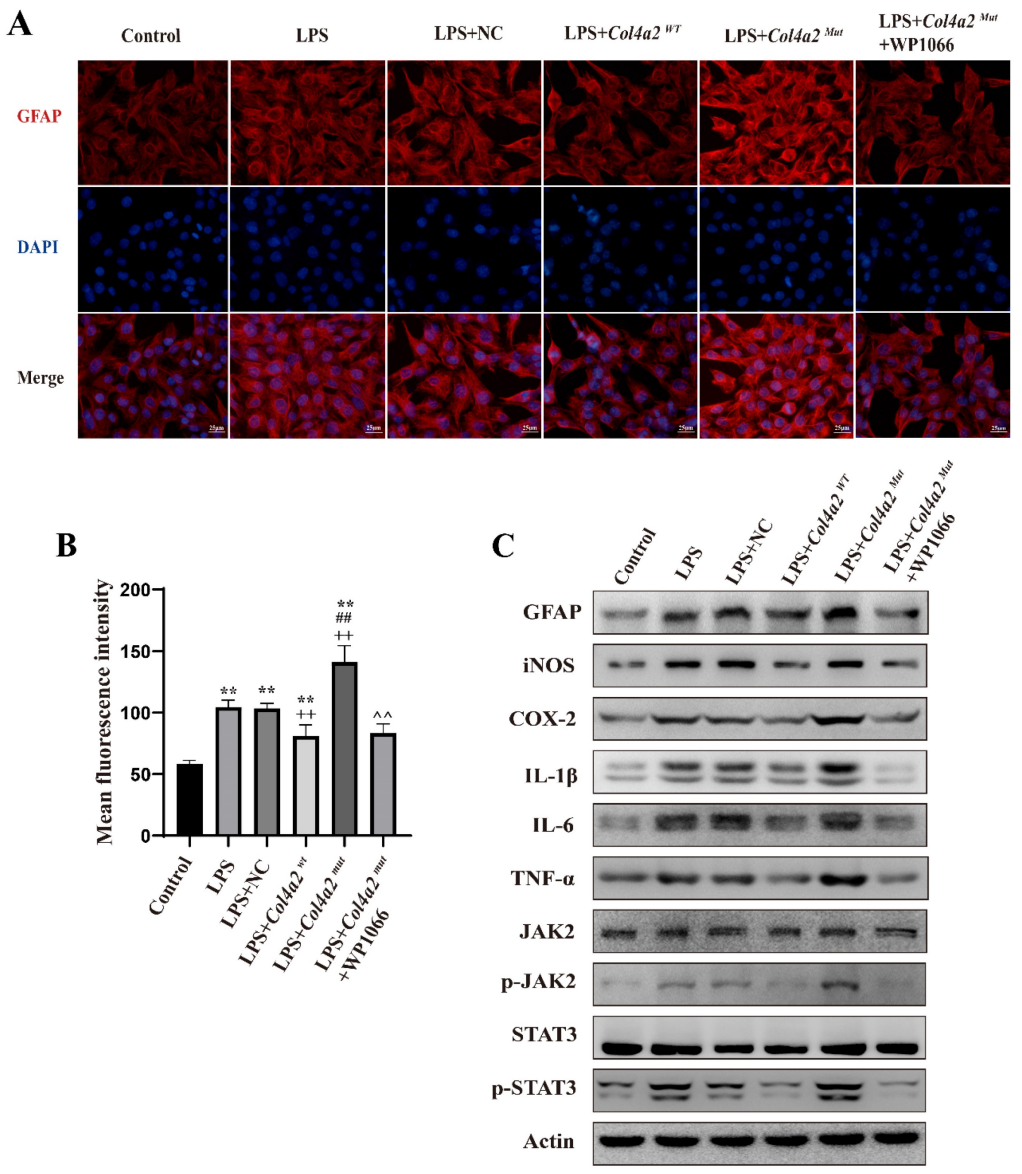
***Col4a2* mutation promotes inflammation and astrocyte activation by stimulating JAK/STAT signaling in CTX-TNA cells.** CTX-TNA cells were stimulated with LPS and transfected with the *Col4a2^Wt^*-AdV vector or the *Col4a2^Mut^*-AdV vector or treated with WP1066 (a JAK2/STAT inhibitor). (A) The fluorescence intensity of GFAP was observed in 6 different groups (50 CTX-TNA cells per group) from 3 repeated experiments. The scale bar represents 25 μm. (B) Quantitative graphs displaying the mean fluorescence intensity of the GFAP-positive cells in (A). The figure includes the results of one-way ANOVA with calculated *p* values. (C) Western blot analysis of GFAP, IL-1β, IL-6, TNF-α, JAK2, p-JAK2, STAT3, and p-STAT3 was performed. The proteins for the western blot analysis were extracted from cell lysates. **: vs. con, p<0.01; ++: vs. LPS+NC, p<0.01; ##: vs. LPS+ *Col4a2^Wt^*, *p*<0.01; ^^: vs. LPS+ *Col4a2^Mut^*, p<0.01.

**Figure 3:**
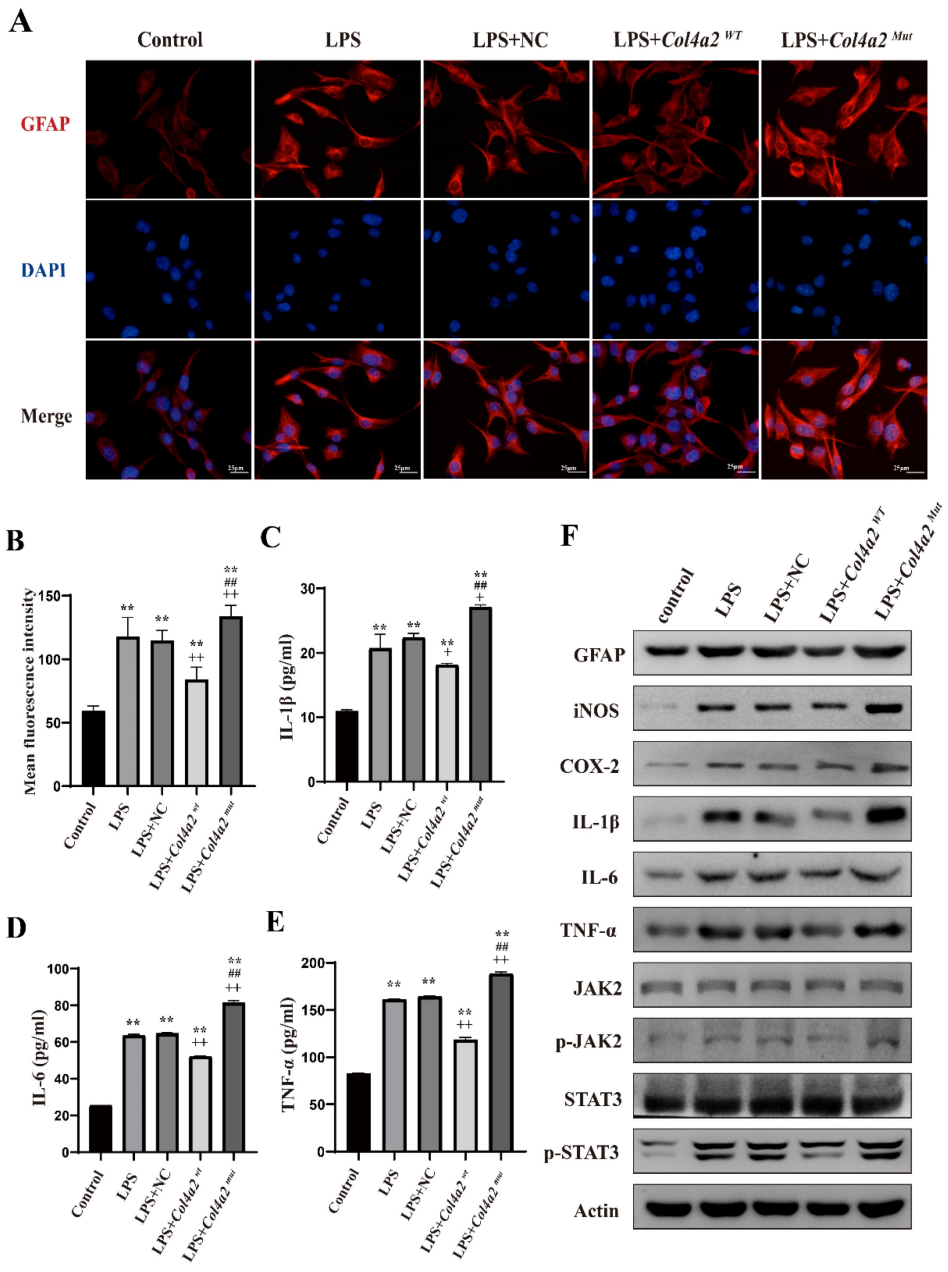
***Col4a2* mutation promotes inflammation and astrocyte activation in primary astrocytes.** Primary astrocytes were stimulated with LPS and transfected with either the *Col4a2^Wt^*-AdV vector or the *Col4a2^Mut^*-AdV vector. (A) The fluorescence intensity of GFAP (red) was measured in 5 different primary astrocyte groups, with 50 cells per group from 3 repeated experiments. The scale bar indicates 25 μm. (B) Quantitative graphs displaying the mean fluorescence intensity of the GFAP-positive cells in (A). (C-E) Enzyme-linked immunosorbent assays were used to measure IL-1β, IL-6, and TNF-α levels in primary astrocyte supernatants from 3 repeated experiments. The figure includes one-way ANOVA results with corresponding *p* values. (F) Western blot analysis was performed for GFAP, IL-1β, IL-6, TNF-α, JAK2, p-JAK2, STAT3, and p-STAT3. The proteins for the western blot analysis were extracted from cell lysates. **: vs. control, p<0.01; ++: vs. LPS+NC, p<0.01; ##: vs. LPS+ *Col4a2^Wt^*, *p*<0.01.

**Figure 4:**
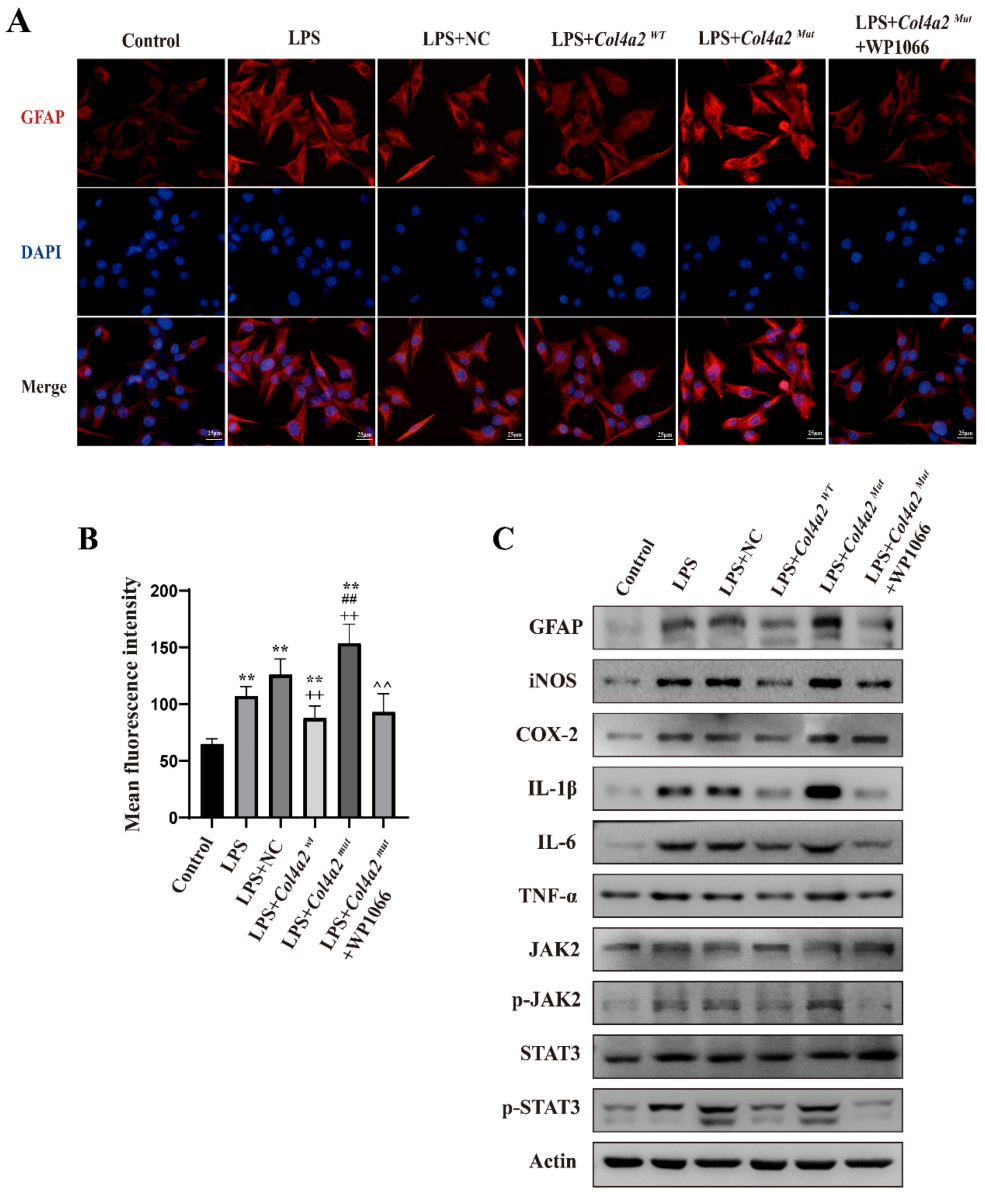
***Col4a2* mutation promotes inflammation and astrocyte activation by stimulating JAK/STAT signaling in primary astrocytes.** Primary astrocytes were stimulated with LPS and transfected with the *Col4a2^Wt^*-AdV vector or the *Col4a2^Mut^*-AdV vector or treated with WP1066 (a JAK2/STAT inhibitor). (A) The fluorescence intensity of GFAP in six different primary astrocyte groups (50 cells per group) from 3 repeated experiments is shown. The scale bar represents 25 μm. (B) Quantitative graphs displaying the mean fluorescence intensity of the GFAP-positive cells in (A). One-way ANOVA with calculated *p* values is presented in the figure. (C) Western blot analysis was conducted for GFAP, IL-1β, IL-6, TNF-α, JAK2, p-JAK2, STAT3, and p-STAT3. The proteins for the western blot analysis were extracted from cell lysates. **: vs. control, p<0.01; ++: vs. LPS+NC, p<0.01; ##: vs. LPS+* Col4a2^Wt^*, *p*<0.01; ^^: vs. LPS+ *Col4a2^Mut^*, p<0.01.

As neuroinflammation was identified in *Col4a2* mutation-associated epilepsy, further investigation into the potential pathways activating the inflammatory response was necessary. The JAK-STAT signaling pathway, known as a common pathway for many inflammatory responses, was also examined. The JAK/STAT signaling pathway was activated in CTX-TNA cells (Fig. 1F, Fig. 2C) and primary astrocytes (Fig. 3F, Fig. 4C) following *Col4a2* mutation, as evidenced by increased phosphorylation of JAK2 and STAT3. However, the total expression levels of JAK2 and STAT3 remained unchanged. Subsequent experiments using the JAK/STAT inhibitor WP1066 revealed that the activation of astrocytes induced by *Col4a2^Mut^* could be reversed by treatment with WP1066 in CTX-TNA cells (IF: *p*<0.001, Fig. 2A-B) and primary astrocytes (IF: *p*<0.001, Fig. 4A-B). Moreover, WP1066 treatment also attenuated the inflammatory responses triggered by *Col4a2^Mut^* in CTX-TNA cells (Fig. 2C). and primary astrocytes (Fig. 4C). Collectively, these findings suggest that *Col4a2* mutation drives astrocyte activation and inflammation through the JAK/STAT pathway.

## Discussion

Epilepsy is a significant chronic neurological disease that is closely related to genetics. The onset and progression of epilepsy involve nerve damage, increased oxidative stress, abnormal glial cell activity, mitochondrial dysfunction, and inflammatory responses.* Missense mutations in Col4a2*, the gene encoding the α2 chain of type IV collagen, are known to be associated with multiple diseases. This study focused on *Col4a2*-related infantile epileptic spasm syndrome and revealed incomplete penetrance of the *Col4a2* gene mutation. Additionally, elevated levels of the cytokines IL-1β and IL-6 were observed in the cerebrospinal fluid of these patients before seizure onset despite the absence of infection. Currently, the functions of developmental and epileptic encephalopathy (DEE)-related genes are categorized into six subtypes according to function: synapsis, neurotransmitters and receptors; signal transduction; ion channels; DNA and RNA; organelles and the cell membrane; and development and growth 21. However, our results suggest that mutations in the *Col4a2* gene may induce an inflammatory microenvironment; thus, the *Col4a2* gene mutation may be classified as a type VII according to the classification of DEE-causing gene functions. Single-cell transcriptomics and epitope sequencing (CITE-seq) of brain tissue from individuals with refractory epilepsy at epileptic foci revealed proinflammatory mechanisms. The immune transcriptome displayed differential expression of several genes, including collagen genes associated with the basement membrane of endothelial cells in neurovascular units. This study aimed to investigate the proinflammatory microenvironment, which is characterized by widespread microglial activation and infiltration of other proinflammatory immune cells, providing insights into refractory epilepsy 22. Previous studies have shown that activated astrocytes can play a vital role in epileptogenesis independent of neurons 23-25. Therefore, we hypothesized that mutations in the *Col4a2* gene could impact astrocyte activity. To investigate the impact of the *Col4a2* mutation on epilepsy, we choose an unreported mutation site* Col4a2^Mut^* (c.1838G>T) and introduced itinto the CTX-CNA astrocyte cell line and primary astrocytes. Compared to those in the *Col4a2^Wt^* group, there were significant increases in GFAP levels in the *Col4a2^Mut^* (c.1838G>T) group. It is widely recognized that increased GFAP expression in the context of epilepsy signifies astrocyte activation. Our findings revealed visible activation of both CTX-CNA and primary astrocytes following *Col4a2* mutation.

Astrocyte activation promotes the release of inflammatory cytokines, which further exacerbates astrocyte activation. Our clinical data indicated that the levels of inflammatory cytokines, including IL-1β and IL-6, are elevated in the cerebrospinal fluid of patients with *Col4a2* mutations. These findings suggest that neuroinflammation may play a role in the development of *Col4a2* mutation-related epilepsy. The type IV collagen produced by this gene contributes to the basement membrane structure, impacting the permeability of the blood‒brain barrier. Additionally, astrocytes are crucial components of the blood‒brain barrier and produce inflammatory cytokines. We also observed increased secretion of proinflammatory cytokines such as iNOS, COX2, IL-1β, IL-6, and TNF-α following *Col4a2* mutation. Both prolonged seizures and astrocyte activation can trigger the release of proinflammatory cytokines, intensifying the inflammatory response. Changes in astrocyte function can disrupt the blood‒brain barrier, leading to further release of proinflammatory cytokines.

The JAK/STAT signaling pathway is a crucial mechanism for regulating the expression of inflammatory genes and immune functions. It is essential for the development of T and B cells, as well as for combating viral and bacterial infections, regulating neurological functions, and contributing to epilepsy. Although there are 4 JAKs and 7 STATs, the pathway mainly involves JAK1 and JAK2 due to the low expression levels of JAK3 and Tyk2 in astrocytes 26, 27. The JAK2/STAT3 signaling pathway is highly conserved and serves as a critical cellular component 28. Therefore, we initially focused on the star pathway JAK2/STAT3. Our study indicated that astrocyte activation and neuroinflammation are significant aspects of epilepsy associated with *Col4a2* mutations. These mutations trigger astrocyte activation and the activation of JAK2/STAT3 signaling, with subsequent release of inflammatory cytokines. Moreover, studies have demonstrated that the activation of JAK2/STAT3 signaling promotes the generation of GFAP-positive astrocytes and mediates inflammatory responses induced by LPS 29-31. Following the phosphorylation of JAK2 and STAT3, phosphorylated STATs combine to form homodimers or heterodimers with other STAT proteins. These complexes then move into the nucleus, where they regulate the transcription of various proinflammatory cytokines 32. Here, we explored mutations within the *Col4a2* gene that enhance JAK/STAT signaling, leading to increased phosphorylation of JAK2 and STAT3. Furthermore, treatment with the JAK/STAT signaling inhibitor WP1066 reduced JAK2 and STAT3 phosphorylation, astrocyte activation, and neuroinflammatory responses in rat primary astrocytes and CTX-TNA cells. Similarly, we observed that overexpression of the *Col4a2^Wt^* gene suppressed LPS-induced astrocyte activation and neuroinflammatory responses, suggesting a potential protective role of the *Col4a2^Wt^* gene in epilepsy.

Our data suggest that an unreported mutation site* Col4a2^Mut^* (c.1838G>T) may play an important role in the development of epilepsy by activating the JAK/STAT pathway to regulate neuroinflammation. However, the specific mechanism by which *Col4a2* mutation triggers JAK/STAT activation remains unclear. In addition, the limitations of our study include the small sample size, and our experimental methods need to be more comprehensive. Further comprehensive studies are needed to address these questions. Our initial investigation into the role of the JAK/STAT pathway in the pathogenesis of *Col4a2* mutation-associated epilepsy has laid a foundation for future research on therapeutic approaches. This pathway could serve as an effective target for new therapies in *Col4a2* mutation-associated epilepsy. Additionally, our study revealed the presence of an inflammatory microenvironment in *Col4a2* mutation-associated DEEs and suggested that this type of epilepsy may be associated with a type VII inflammatory microenvironment according to the functional classification of genes causing DEEs.

While the exact source of proinflammatory cytokine production remains fully elucidated, we have formulated a hypothesis regarding the mechanism involved mutated *Col4a2*. The endoplasmic reticulum is a crucial site for protein synthesis in eukaryotic cells. When protein synthesis is excessive or if proteins cannot be adequately folded and transported, proteins build up in the endoplasmic reticulum, leading to endoplasmic reticulum stress and triggering unfolded protein reactions. Previous studies have indicated that endoplasmic reticulum stress plays a role in the development of epilepsy and can result in neuroinflammation before seizures occur 4. Activation of the PERK signaling pathway during endoplasmic reticulum stress promotes inflammation and contributes to neuronal damage in epilepsy 33. As a result, endoplasmic reticulum stress has emerged as a significant factor in neuroinflammation associated with genetic epilepsy, independent of epileptic seizures.

Type IV collagen deposition can lead to chronic endoplasmic reticulum stress 34, 35. Following a mutation in *Col4a2*, defects in the vascular basement membrane were observed, along with high levels of *Col4a2* mutant protein retention in the endoplasmic reticulum, leading to endoplasmic reticulum stress and activation of the unfolded protein response 36. Research has also shown that the accumulation of mutated proteins can induce endoplasmic reticulum stress prior to epileptic seizures in genetic epilepsy, thereby promoting neuroinflammation. Astrocytes have been found to express all initial sensors of the unfolded protein response and are sensitive to endoplasmic reticulum stress-inducing molecules 37. Our study indicates that mutations in *Col4a2* can trigger astrocyte activation and neuroinflammation in epilepsy through the JAK/STAT pathway. Therefore, it is reasonable to speculate that endoplasmic reticulum stress induction may be the source of neuroinflammation. This will be our next primary research direction, which is a long and complicated research process.

## Conclusion

This study revealed incomplete penetrance of an unreported mutation site* Col4a2^Mut^* (c.1838G>T) and elevated levels of the cerebrospinal fluid cytokines IL-1β and IL-6 in infants with *Col4a2*-related epileptic spasm syndrome. The vast majority of known DEE-causing genes can be categorized into six subtypes. Our results suggest that an inflammatory microenvironment may be induced by mutations in *Col4a2*, which could be categorized as type VII in the functions classification of genes causing DEE. Notably, neuroinflammation and astrocyte activation are vital features of epilepsy linked to *Col4a2* mutations and may be regulated via the JAK/STAT signaling pathway. This research lays a foundation for the development of new treatments for DEEs that target neuroinflammation and astrocytes.

## Funding

This work was supported by Startup Fund for scientific research, Fujian Medical University (Grant number: 2023QH1219) and Science and Technology Innovation Fund of Fujian Children's Hospital (Children YCXY202401).

## Figures and Tables

**Table 1:** Summary of the phenotypic features of our 8 patients

P	Current age, Sex	Seizure onset age	Seizure semiology	Other phenotypes	Developmental delay	EEG	MRI	ASMs tried	Effective ASMs	Seizure outcome
1	3.5 y, M	3 m	ES	None	Severe → Moderate	H → Multifocal	N → N	P	P	Controlled; Seizure-free for 2 y 9 m
2	4 y, F	4 m	ES	Dystonia	Severe → Severe	Multifocal → Multifocal	WG → N	VPA LEV TPM OXC LCM LTG CZP ACTH P KD	None	Uncontrolled
3	5 y, M	4 m	ES	None	Severe → Moderate	Multifocal → N	N → N	ACTH P VGB	ACTH P VGB	Controlled; Seizure-free for 4 y
4	2 y 2 m, M	7 m	ES	None	Severe → Moderate	H → Multifocal	N → N	ACTH VGB	VGB	Controlled; Seizure-free for 1 y
5	7 y, F	7 m	ES, FS, T	None	Severe → Severe	Multifocal → Multifocal	N → N	ACTH VGB ZNS CZP CLB LTG VPA TPM LEV LCM OXC KD	None	Uncontrolled
6	8 y, M	8 m	ES, FS, T	Nystagmus	Severe → Severe	H → Multifocal	N → N	PB VPA TPM LEV OXC ACTH P VGB CZP PER KD VNS	None	Uncontrolled
7	3 y 4 m, M	10 m	ES	None	Severe → Severe	H → Multifocal	WG → N	TPM VPA P LEV CZP CLB KD TCZ	TCZ	Uncontrolled
8	18 y, M	9 m	ES	None	Moderate → Mild	Multifocal → N	N → N	P	P	Controlled; Seizure-free for 16 y

Abbreviations: P: patient; y: year/year; m: month/month; M: male; F: female; ASMs: anti-seizure medications; EEG: electroencephalogram; ES: epileptic spasms FS: focal seizures; T: tonic seizures; N: normal; H: hypsarrhythmia; Multifocal: multifocal sharp waves, spike waves, sharp slow waves or spike slow waves; P: prednisone; VPA: valproate acid; LEV: levetiracetam; TPM: topiramate; OXC: oxcarbazepine; LCM: lacosamide; LTG: lamotrigine; CZP: clonazepam; KD: ketogenic diet; VGB: vigabatrin; ZNS: zonisamide; CLB: clobazam; PB: phenobarbital; PER: perampanel; VNS: vagus nerve stimulation; TCZ: tocilizumab; WG: widened gap in the extracerebral space

**Table 2:** Summary of the genetic findings of our 8 patients

P	Coordinates	Gene	Base change	Amino acid change	Predicted effect on protein	Zygosity	Inheritance	NM	Interpretation	Novel/ Reported
1	Chr13: 111102095	*COL4A2*	c.1148C>T	p.P383L	Missense	Heterozygous	De novo	NM_001846.4	Likely pathogenic	R
2	Chr13: 111147740	*COL4A2*	c.3686G>A	p.G1229D	Missense	Heterozygous	De novo	NM_001846.4	Likely pathogenic	N
3	Chr13: 111134925	*COL4A2*	c.2821G>A	p.G941R	Missense	Heterozygous	De novo	NM_001846.4	Likely pathogenic	R
4	Chr13: 111082290	*COL4A2*	c.536G>A	p.R179H	Missense	Heterozygous	De novo	NM_001846.4	Likely pathogenic	N
5	Chr13: 111114690	*COL4A2*	c.1735C>T	p.R579C	Missense	Heterozygous	De novo	NM_001846.4	Likely pathogenic	N
6	Chr13: 111099154	*COL4A2*	c.1021G>A	p.G341R	Missense	Heterozygous	De novo	NM_001846.4	Likely pathogenic	R
7	Chr13: 111117813	*COL4A2*	c.1838G>T	p.G613V	Missense	Heterozygous	Mother	NM_001846.4	Uncertain significance	N
8	Chr13: 111156194	*COL4A2*	c.4139G>A	p.G1380D	Missense	Heterozygous	De novo	NM_001846.4	Likely pathogenic	N

Abbreviations: P: patient; Chr: chromosome; N: novel, which means this mutation has not been reported; R: reported, which means this mutation has been reported.

**Table 3:** Levels of cerebrospinal fluid cytokines before seizure-free status in our 6 patients

P	IL-1β (0-12.1)	IL-2 (0-11.4)	IL-4 (0-12.9)	IL-5 (0-3.4)	IL-6 (0-20.0)	IL-8 (0-21.4)	IL-10 (0-5.9)	IL-12p70 (0-3.2)	IL-17 (0-20.6)	IFN-α (0-7.9)	IFN-γ (0-17.3)	TNF-α (0-5.5)
2	42.3	2.5	1.8	1.5	47.9	11.2	3.5	2.5	7.5	3.4	4.7	2.3
3	10.5	2.1	2.5	1.8	34.6	9.5	2.3	2.1	8.1	2.9	6.2	3.5
4	25.9	3.3	3.2	1.3	18.7	6.3	2.5	2.2	5.2	4.1	5.9	3.4
5	36.9	2.8	4.6	2.4	59.8	4.2	3.1	1.6	4.1	4.5	7.1	1.9
6	33.1	4.3	2.1	2.7	49.2	3.1	1.8	1.8	3.6	5.2	8.3	3.2
7	44.7	2.2	5.1	2.2	60.5	14.2	2.4	1.9	8.4	2.5	4.5	2.9

Abbreviations: P = patient. Cerebrospinal fluid cytokine levels are reported in pg/ml. The normal reference ranges of the cytokines are shown in parentheses.

**Table 4:** Levels of cerebrospinal fluid cytokines after multiple treatments in our 6 patients

P	IL-1β (0-12.1)	IL-2 (0-11.4)	IL-4 (0-12.9)	IL-5 (0-3.4)	IL-6 (0-20.0)	IL-8 (0-21.4)	IL-10 (0-5.9)	IL-12p70 (0-3.2)	IL-17 (0-20.6)	IFN-α (0-7.9)	IFN-γ (0-17.3)	TNF-α (0-5.5)
3	5.9	2.1	2.5	1.8	9.1	9.5	2.3	2.1	8.1	2.9	6.2	3.5
4	3.5	3.3	3.2	1.3	6.3	6.3	2.5	2.2	5.2	4.1	5.9	3.4
5	25.2	2.8	4.6	2.4	49.7	4.2	3.1	1.6	4.1	4.5	7.1	1.9
7	32.3	2.2	5.1	2.2	25.4	14.2	2.4	1.9	8.4	2.5	4.5	2.9

Abbreviations: P = patient. Cerebrospinal fluid cytokine levels are reported in pg/ml. The normal reference ranges of the cytokines are shown in parentheses.
